# The Ontology of the Amphioxus Anatomy and Life Cycle (AMPHX)

**DOI:** 10.3389/fcell.2021.668025

**Published:** 2021-04-26

**Authors:** Stephanie Bertrand, João E. Carvalho, Delphine Dauga, Nicolas Matentzoglu, Vladimir Daric, Jr-Kai Yu, Michael Schubert, Hector Escrivá

**Affiliations:** ^1^CNRS, Biologie Intégrative des Organismes Marins, Sorbonne Université, Paris, France; ^2^CNRS, Laboratoire de Biologie du Développement de Villefranche-sur-Mer, Institut de la Mer de Villefranche, Sorbonne Université, Paris, France; ^3^Bioself Communication, Marseille, France; ^4^European Bioinformatics Institute (EMBL-EBI), Cambridge, United Kingdom; ^5^Institute of Cellular and Organismic Biology, Academia Sinica, Taipei City, Taiwan; ^6^Marine Research Station, Institute of Cellular and Organismic Biology, Academia Sinica, Yilan, Taiwan

**Keywords:** amphioxus (lancelet), ontology (ontologie), cephalochordates, evodevo model organisms, life cycle, anatomy

## Abstract

An ontology is a computable representation of the different parts of an organism and its different developmental stages as well as the relationships between them. The ontology of model organisms is therefore a fundamental tool for a multitude of bioinformatics and comparative analyses. The cephalochordate amphioxus is a marine animal representing the earliest diverging evolutionary lineage of chordates. Furthermore, its morphology, its anatomy and its genome can be considered as prototypes of the chordate phylum. For these reasons, amphioxus is a very important animal model for evolutionary developmental biology studies aimed at understanding the origin and diversification of vertebrates. Here, we have constructed an amphioxus ontology (AMPHX) which combines anatomical and developmental terms and includes the relationships between these terms. AMPHX will be used to annotate amphioxus gene expression patterns as well as phenotypes. We encourage the scientific community to adopt this amphioxus ontology and send recommendations for future updates and improvements.

## Introduction

Cephalochordates (i.e., amphioxus) are a group of benthic marine filter feeding animals which live buried in the sand of shallow coastal environments in most temperate and tropical seas. Although there are only about thirty species and three genera (*Branchiostoma*, *Epigonichthys*, and *Asymmetron*), this chordate subphylum attracts the attention of numerous researchers because of its key phylogenetic position, representing the earliest diverging evolutionary lineage within chordates, and for its extraordinary morphological, anatomical, and genomic conservation with the last common ancestor of all chordates, including vertebrates (Bertrand and Escriva, 2011; Escriva, 2018). Some of these conserved morphological characters include a dorsal hollow neural tube and a dorsal notochord, pharyngeal slits, segmented muscles, and gonads as well as organs homologous to those of vertebrates, such as the pronephric kidney or an endostyle. However, some vertebrate-specific structures or organs are absent from amphioxus. These include paired sensory organs (eyes and ears), limbs or migrating neural crest cells. At the genomic level, amphioxus is also vertebrate-like but simpler, since their genomes did not experience the whole genome duplications that occurred in the vertebrate lineage (Dehal and Boore, 2005; Putnam et al., 2008). In addition, the amphioxus genome also shows simplified 3D structure and genetic regulation when compared to vertebrate genomes (Acemel et al., 2016; Marletaz et al., 2018).

**FIGURE 1:**
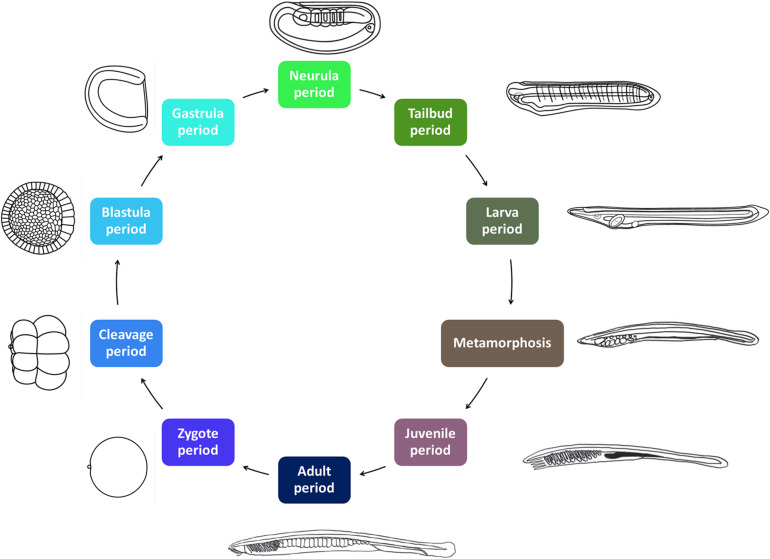
The amphioxus life cycle. The ten developmental periods defined in AMPHX are indicated. Periods between zygote and metamorphosis are planktonic. The juvenile and adult periods are benthic. The size of the *Branchiostoma lanceolatum* embryos and larvae ranges from 80 to 100 μm in the zygote and early embryonic stages to several millimeters in the larval stages. Metamorphosis occurs in larvae with sizes of between 5 and 7 mm. Juveniles range in size from 5–7 mm to 3 cm and adults from 3 to 8 cm. Illustrations from zygote to larva periods have been adapted from Carvalho et al. (2021).

All amphioxus species are gonochoric, and reproduce by external fertilization. Amphioxus males and females release their gametes in the water column during the spawning season, which, depending on the species, spans 3–6 months every year, usually during spring and summer time (Bertrand and Escriva, 2011). Amphioxus have a typical bentho-pelagic life cycle (Figure 1). An embryonic period is followed by a larval period, which ends when the larva undergoes metamorphosis, which, depending on the species, takes place from a few weeks to several months after fertilization. While amphioxus embryos and larva are planktonic, the juveniles emerging after metamorphosis are benthic and live buried into the substrate. The morphology of the post-metamorphic juvenile is identical to that of the adult. However, juveniles need to grow for a period of time, which can range from a few weeks in tropical species to several years in temperate species, before they start developing gonads and thus become sexually mature adults (Bertrand and Escriva, 2011).

Due to their phylogenetic position and extreme conservation, many laboratories around the world are interested in the study of the mechanisms controlling the embryonic development of amphioxus as well as in other aspects of its biology, such as regenerative capacities or its immune system (Somorjai et al., 2012; Yuan et al., 2015; Holland and Somorjai, 2020). These studies are mainly carried out on three or four species of the genus *Branchiostoma*, but recently also on a species of the genus *Asymmetron* (Holland and Holland, 2010). For this reason, and in order to standardize the results obtained in different species, we established a universal staging system (Carvalho et al., 2021) as well as an ontology of the development and anatomy of this chordate subphylum. This ontology, AMPHX, is the first one developed for a cephalochordate and follows the example of ontologies previously established for other chordate species (Segerdell et al., 2008; Manni et al., 2014; Van Slyke et al., 2014; Hotta et al., 2020). The AMPHX ontology has been conceived as an open and implementable automated retrieval system that can be integrated with biological information, such as gene expression or phenotypes. We hope that this ontology will be improved and updated whenever new studies on amphioxus appear in the literature.

## Materials and Methods

The different AMPHX terms, synonyms, definitions, and information on embryonic development and anatomical structures have been obtained from textbooks, journal articles, and scientific observations. This information has been compiled and formatted in two table files (Supplementary Tables 1, 2): one for life cycle and one for anatomy terms. The anatomy ontology was built following a bottom-up approach, starting from the cells and anatomical structures present in early stages of development, gradually introducing additional terms as new structures appear during embryogenesis, metamorphosis, and during juvenile and adult periods. We used the Webulous server^1^ to create spreadsheets with an initial set of terms and relationships for the ontology. The resulting spreadsheet was integrated into an ontology development kit (ODK) workflow, using ROBOT (Jackson et al., 2019) templates, which we used for managing the transformation of our spreadsheets into the Web ontology language (OWL). We manage our ontology curation as well as collaborative and release workflows through GitHub^2^.

## Results

### Ontology Access

In the AMPHX ontology, we have described the anatomy and development, from the oocyte to the adult, of the cephalochordate amphioxus (i.e., the *Branchiostoma* genus). AMPHX can be downloaded from the AMPHX GitHub repository (see text footnote 2). The ontology was officially accepted into the OBO foundry and is listed in the OBO Foundry portal^3^ (Smith et al., 2007) and the Ontobee database^4^ (Ong et al., 2017). Users can also browse AMPHX at the ontology lookup service (OLS) at EMBL-EBI^5^ (Côté et al., 2008; Jupp et al., 2015) and Bioportal^6^ (Noy et al., 2009). In order for our data to be consistent with the FAIR principle^7^, we submitted the AMPHX ontology to the FAIRsharing repository (Sansone et al., 2019), which approved our entry^8^.

### Current Content

**FIGURE 2:**
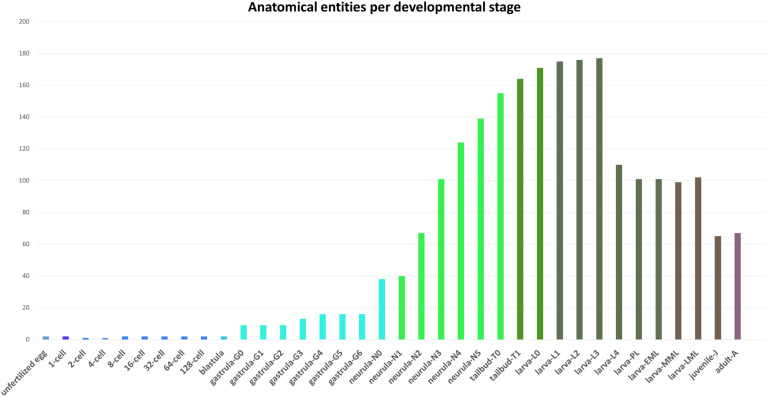
Number of anatomical entities per developmental stage of *Branchiostoma lanceolatum*. The number of anatomical entities (Y-axis) in the unfertilized egg as well as at each developmental stage from the 1-cell stage to the adult stage (X-axis) is indicated.

**FIGURE 3:**
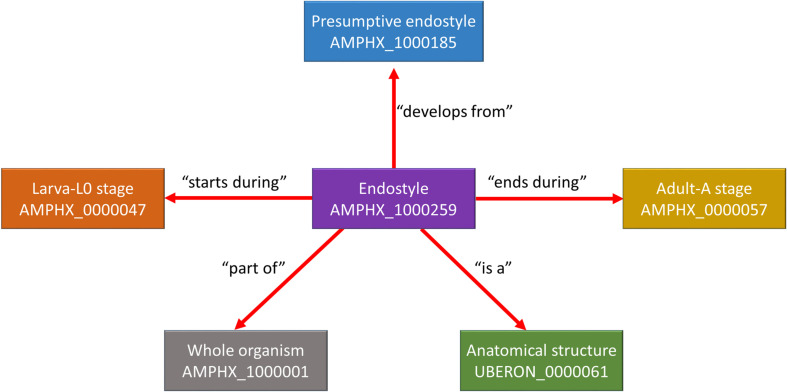
Example for the representation of a structure-based subclass hierarchy in the amphioxus ontology (AMPHX). Using the amphioxus endostyle as an example, the partonomy relationships between different terms (“part_of”, “develops_from”, and “is a”) as well as the developmental stages during which the structure is present (“starts during” and “ends during”) are shown. The figure has been adapted from the Graph view utility proposed at OLS (Côté et al., 2008).

The AMPHX ontology tree can be divided into two groups: a developmental entity, from AMPHX:0000001 to AMPHX:0000058 (Supplementary Table 1), and an anatomical entity, from AMPHX:1000001 to AMPHX:1000342 (Supplementary Table 2). In addition, there are three general terms from the cell ontology (CL^9^), corresponding to the following three major classes: cellular component (GO:0005575), cell (CL:0000000), and anatomical structure (UBERON:0000061), with a total number of 403 terms (Figure 2). The AMPHX ontology follows a structure-based subclass hierarchy including a partonomy relation between different terms (“part_of”, BFO_0000050), a developmental hierarchy (“develops_from”, RO_0002202), and a global developmental presence of a given term (“existence_starts_during” and “existence_ends_during”, RO_0002488 and RO_0002492, respectively). The developmental hierarchy and presence terms are equivalent to the relation ontology^10^ (RO). In the structure of AMPHX, each anatomical entity is thus defined by terms describing: being a type of, being a part of, developing from, and existing at (Figure 3).

One of the main aims of the AMPHX is to provide a queryable reference source for information about wild-type amphioxus development and anatomy. For this reason, we have added textual definitions to over 17% of the classes, based on scientific evidence derived from experimental observations. The overall number of these definitions will increase over time, with novel results and data that can be added to the AMPHX ontology.

Integration of ontologies at successive stages was achieved *via* lineage links between mother and daughter territories. 323 such lineage links (“develops_from”) were established, corresponding to 80% of the classes. This level of lineage information is vastly superior to the repertoires of ontologies of other model organisms, such as the *Caenorhabditis elegans* Gross Anatomy Ontology with 0,5% of classes with lineage information (Lee and Sternberg, 2003), the Zebrafish Anatomy and Development Ontology with 17% of classes with lineage information (Van Slyke et al., 2014) or the Xenopus Anatomy Ontology with 38% of classes with lineage information (Segerdell et al., 2008).

The AMPHX development ontology is based on *in vivo* observations [see Carvalho et al. (2021) for a morphological description of the different developmental stages] and is also inspired by the staging system proposed by Hirakow and Kajita (1990, 1991, 1994), which we have expanded by adding and redefining some developmental stages. AMPHX thus presents amphioxus development subdivided into ten different periods from zygote to adult (Figure 1 and Table 1). We first defined the development ontology and subsequently added the anatomy ontology by carefully annotating anatomical structures for each developmental stage, based on information found in the literature. This allowed us not only to include all previously defined cell types, tissues, and organs but also to follow their differentiation through development. This led us to define 342 anatomical terms and assign a specific ID to each one of them.

**TABLE 1:** Amphioxus developmental periods and stages from the zygote to the adult.

Period	Stage	ID	Description
Zygote period		AMPHX:0000008	
	1-cell stage	AMPHX:0000022	Fertilized egg before first cell division
Cleavage period		AMPHX:0000009	
	2-cell stage	AMPHX:0000023	
	4-cell stage	AMPHX:0000024	
	8-cell stage	AMPHX:0000025	
	16-cell stage	AMPHX:0000026	
	32-cell stage	AMPHX:0000027	
	64-cell stage	AMPHX:0000028	
	128-cell stage	AMPHX:0000029	
Blastula period		AMPHX:0000010	
	Blastula stage	AMPHX:0000031	Initiation of asynchronous cell division
Gastrula period		AMPHX:0000011	
	Gastrula-G0 stage	AMPHX:0000032	Initial flattening of the vegetal zone
	Gastrula-G1 stage	AMPHX:0000033	Flattened vegetal pole
	Gastrula-G2 stage	AMPHX:0000034	Invaginated vegetal pole, mesendoderm does not touch ectoderm
	Gastrula-G3 stage	AMPHX:0000035	Cap shaped, mesendoderm touches ectoderm
	Gastrula-G4 stage	AMPHX:0000036	Cup shaped
	Gastrula-G5 stage	AMPHX:0000037	Vase shaped, due to body elongation during blastopore closure
	Gastrula-G6 stage	AMPHX:0000038	Bottle shaped, flattening neuroectoderm
Neurula period		AMPHX:0000012	
	Neurula-N0 stage	AMPHX:0000040	Neural plate, just before blastopore closure
	Neurula-N1 stage	AMPHX:0000041	1–3 somite pairs
	Neurula-N2 stage	AMPHX:0000042	4–5 somite pairs, hatching
	Neurula-N3 stage	AMPHX:0000043	6–7 somite pairs
	Neurula-N4 stage	AMPHX:0000044	8–9 somite pairs, prior to schizocoelic somite formation
	Neurula-N5 stage	AMPHX:0000058	10–11 somite pairs
Tailbud period		AMPHX:0000013	
	Tailbud-T0 stage	AMPHX:0000045	12 somite pairs, tailbud shape, and enlarged pharyngeal region
	Tailbud-T1 stage	AMPHX:0000046	13 somite pairs, mouth and pre-oral pit anlagen, and first pigment spot
Larva period		AMPHX:0000015	
	Larva-L0 stage	AMPHX:0000047	Open mouth, no open gill slits
	Larva-L1 stage	AMPHX:0000048	Open mouth, 1 open gill slit
	Larva-L2 stage	AMPHX:0000049	Open mouth, 2 open gill slits
	Larva-L3 stage	AMPHX:0000050	Open mouth, 3 open gill slits
	Larva-L4 stage	AMPHX:0000051	Open mouth, 4–13 gill slits
	Larva-PL stage	AMPHX:0000052	Open mouth, 14–15 gill slits
Metamorphosis		AMPHX:0000016	
	Larva-EML stage	AMPHX:0000053	Metapleural folds start to grow
	Larva-MML stage	AMPHX:0000054	Gill slit row starts to duplicate, hepatic cecum starts to develop
	Larva-LML stage	AMPHX:0000055	Mouth migration to frontal position
Juvenile period		AMPHX:0000018	
	Juvenile-J stage	AMPHX:0000056	Adult-like morphology, length of less than 3 cm
Adult period		AMPHX:0000020	
	Adult-A stage	AMPHX:0000057	Adult-like morphology, length of more than 3 cm

*A short description of the stages is included. The descriptions have been made using Branchiostoma lanceolatum. This table needs to be adapted slightly when applied to other species (especially in the late neurula and premetamorphic stages, as the number of somites and/or gill slits, the main bases for defining the stages, varies between species).*

### The Development Ontology of *Branchiostoma* sp.

#### Embryonic Development From the Zygote to the Tailbud Stages

The early embryonic development is subdivided into four developmental periods: zygote period (AMPHX:0000008), cleavage period (AMPHX:0000009), blastula period (AMPHX:0000010), and gastrula period (AMPHX:0000011) (Figure 1 and Table 1).

The zygote period comprises only one developmental stage, the 1-cell stage (AMPHX:0000022), which corresponds to the fertilized egg and ends with the first cell division. The cleavage period comprises the first cell divisions, which occur synchronously. This period includes seven developmental stages, from the 2-cell to the 128-cell stage (from AMPHX:0000023 to AMPHX:0000029). The first asynchronous cell divisions mark the start of the blastula period, which includes a single developmental stage, the blastula stage (AMPHX:0000031). The gastrula period includes seven developmental stages: the gastrula-G0 stage through the gastrula-G6 stage (AMPHX:0000032 to AMPHX:0000038). The gastrula-G0 stage corresponds to the initial flattening of the vegetal zone of the embryo. The gastrula-G1 stage corresponds to the stage at which the vegetal pole is flattened. During the gastrula-G2 stage, the vegetal pole is invaginating, but the mesendoderm does not touch the ectoderm. As soon as the mesendoderm touches the ectoderm, the embryo reaches the gastrula-G3 stage, also called cap-shaped gastrula. The gastrula-G4 stage corresponds to the cup-shaped gastrula. At the gastrula-G5 stage, the gastrula starts to elongate during blastopore closure, forming the vase-shaped gastrula. The final gastrula stage, the gastrula-G6 stage, corresponds to a bottle-shaped gastrula. At this stage, the neuroectoderm starts to flatten [for a morphological description of these stages see Carvalho et al. (2021)].

The gastrula period is followed by two additional periods of embryonic development, the neurula period (AMPHX:0000012), which immediately follows the gastrula period, and the tailbud period (AMPHX:0000013), which immediately follows the neurula period. Neurulation starts at the neurula-N0 stage (AMPHX:0000040) when the neural plate is visible, the blastopore is just about to close, and no somites have formed yet. The neurula stages following the neurula-N0 stage have been defined according to the number of somites that have developed in the embryo. Thus, the neurula-N1 stage embryo (AMPHX:0000041) has 1–3 somite pairs, and the neurula-N2 stage embryo (AMPHX:0000042) 4–5 somite pairs. The neurula-N2 stage is further the stage during which the embryo hatches. The last enterocoelic somites form during the neurula-N3 (AMPHX:0000043) and -N4 (AMPHX:0000044) stages characterized by, respectively, 6–7, and 8–9 somite pairs. The neurula-N5 stage (AMPHX:0000058) has 10–11 somite pairs and is characterized by the formation of the first schyzozoelic somites. The neurula-N5 stage marks the end of the neurula period, which is followed by the tailbud period. This period is composed of only two developmental stages, the tailbud-T0 stage (AMPHX:0000045) and the tailbud-T1 stage (AMPHX:0000046). During the tailbud-T0 stage, the twelfth somite pair is formed, and the embryo adopts a tailbud shape with an enlarged pharyngeal region. During the tailbud-T1 stage, the thirteenth somite pair is formed and the mouth and pre-oral pit anlagen are formed as well as the first pigment spot.

#### Post-embryonic Development From the Larva to the Adult

Embryonic development in amphioxus is considered to be completed as soon as the mouth opens, which is when the larva period commences (AMPHX:0000015). We have defined most of the larval stages according to the number of formed pharyngeal slits. However, from the moment when the first three pharyngeal slits have formed and until the initiation of metamorphosis, there are only very few anatomical changes in the larva (except for the successive addition of new pharyngeal slits). We have thus defined only a single developmental stage for amphioxus larvae with four to thirteen gill slits. In total, the larva period comprises six developmental stages, starting with larva-L0 stage (AMPHX:0000047), which is characterized by the opening of the mouth. The larva-L0 stage is followed by the larva-L1 (AMPHX:0000048), -L2 (AMPHX:0000049), and -L3 (AMPHX:0000050) stages, during which, respectively, the first, second, and third pharyngeal slits appear. Subsequently, during the larva-L4 stage (AMPHX:0000051), new pharyngeal slits are added sequentially until the thirteenth slit. This stage is followed by the final larva stage, the larva-PL stage (AMPHX:0000052). This premetamorphic larva is characterized, in *Branchiostoma lanceolatum*, by 14–15 pharyngeal slits.

Metamorphosis in amphioxus is an extremely complex process where new structures appear and others are reorganized (Paris et al., 2008). We have divided the metamorphosis period (AMPHX:0000016) into three stages, based on the most drastic anatomical changes. The first anatomical change, which is initiated at the beginning of metamorphosis, is the appearance of the metapleural folds on both sides of the body of the early metamorphic larva. This appearance of the metapleural folds marks the larva-EML stage (AMPHX:0000053). The subsequent mid-metamorphic larva, referred to as the larva-MML stage (AMPHX:0000054), is characterized by the duplication of the row of pharyngeal slits and the formation of the hepatic cecum. The metamorphic process is completed when the frontal migration of the mouth has occurred in the late-metamorphic larva at the larva-LML stage (AMPHX:0000055).

Following metamorphosis, the juvenile period (AMPHX:0000018) commences. This period comprises only one developmental stage, the juvenile-J stage (AMPHX:0000056). Juveniles show an adult-like morphology and anatomy but lack gonads. In *B. lanceolatum*, the presence of mature gonads has never been observed in animals that have not reached a length of at least 3 cm. Therefore, we considered that the juvenile-J stage includes animals that have completed metamorphosis, but have not reached a length of 3 cm.

The last developmental period of the amphioxus life cycle is the adult period (AMPHX:0000020), which only contains one stage, the adult-A stage (AMPHX:0000057), and refers to post-metamorphic animals with a size of over 3 cm.

In addition to these different developmental periods and stages, we have included in AMPHX another non-developmental period, the gametogenesis period (AMPHX:0000001), which includes both oogenesis and spermatogenesis (AMPHX:0000002 and AMPHX:0000003, respectively) as well as a non-developmental stage, the gamete stage (AMPHX:0000004). This gamete stage is composed of the unfertilized egg stage (AMPHX:0000005) and the spermatozoid stage (AMPHX:0000006).

### The Amphioxus Anatomy Ontology

The AMPHX anatomy ontology uses three high level terms from the cell ontology (CL, see text footnote 9): cell (CL:0000000), cellular component (GO:0005575), and anatomical structure (UBERON:0000061). Moreover, we have defined amphioxus-specific terms to define the type of a structure (i.e., “is a”). These terms include for example “whole organism” (AMPHX:1000001), “embryonic territory” (AMPHX:1000005), and “tissue” (AMPHX:1000004). When homologous terms exist in other ontologies, we have included these terms as a subclass (“similar to”). For example, the term “blastomere” (AMPHX:1000002) is similar to the NCI Thesaurus OBO Edition term NCIT:C12518, defined as “a cell formed by cleavage division during embryogenesis”, the term “zygote” (AMPHX:1000013) is similar to the identical term in the Spider Ontology SPD:0000786 and is defined as “diploid cell produced by the fusion of sperm cell nucleus and egg”, and the term “presumptive neural plate” (AMPHX:1000041) is similar to the identical term in the Uber-anatomy ontology (Mungall et al., 2012) UBERON:0007284 and is defined as “a presumptive structure that has the potential to develop into a neural plate”.

All anatomical entities annotated in the AMPHX ontology were analyzed during ontogeny of *Branchiostoma* sp., stage by stage, from the unfertilized egg to the adult. The early developmental stages contain a lower number of anatomical entities, which increase as development proceeds (Figure 2). Thus, the number of entities associated with the developmental stages between the zygote period and the blastula period is 20, with the gastrula period 88, with the neurula period 509, with the tailbud period 319, and with the larva period 1,212. The number of associated entities decreases in the juvenile and adult periods. This effect can be explained by a relative paucity of information, in the scientific literature, on anatomical structures in amphioxus juveniles and adults.

**FIGURE 4:**
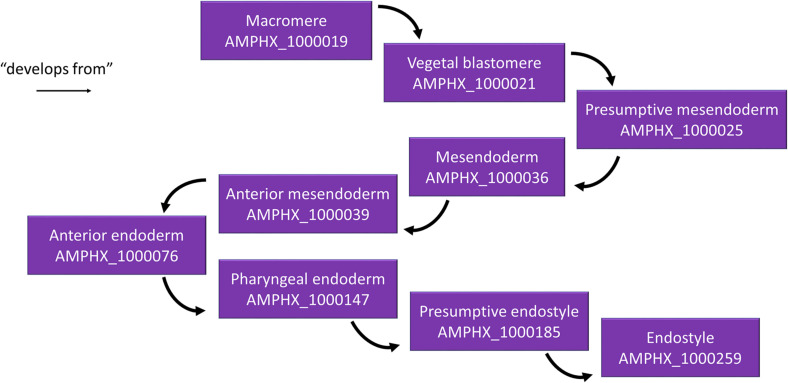
Example for the hierarchical relationships in the AMPHX ontology. Using the amphioxus endostyle as an example, the anatomical entities giving rise to the structure (“develops from”) are indicated, starting with the macromere in the 8-cell stage embryo. The figure has been adapted from the Graph view utility proposed at OLS (Côté et al., 2008).

Different relationships of a given AMPHX term can be analyzed using the graphics tool proposed in OLS (see text footnote 5) (Côté et al., 2008; Jupp et al., 2015). For example, an anatomical entity, such as the endostyle (AMPHX:1000259), which is similar to the term UBERON:0006870, defined as “a longitudinal ciliated groove on the ventral wall of the pharynx which produces mucus to gather food particles”, can be represented as a function of its hierarchical relationships, including “is a”, “develops from,” and “part of”, as well as of its development (“existence starts during”, “existence ends during”) (Figure 3). This representation can be expanded to show its complete development from the earliest cell types, tissues or organs (Figure 4). Explanations and a visual demonstration of the use of OLS, and in particular the use of the graphics tool, can be viewed on Youtube^11^.

## Discussion

The AMPHX ontology has been designed to describe the anatomy and the development of amphioxus during its whole life cycle according to the principles of the open biomedical ontologies (OBO) Foundry (Smith et al., 2007). The AMPHX ontology will allow users to search and identify both developmental stages and anatomical structures and to follow the complete ontogeny of organs or structures of interest from the zygote to the adult. While the terms defining the development life cycle stages in AMPHX (from AMPHX:0000001 to AMPHX:00000058) can be directly applied to all amphioxus genera (i.e., *Branchiostoma*, *Epigonichthys*, and *Asymmetron*), the terms defining the anatomy (from AMPHX:1000001 to AMPHX:1000342) have been obtained from and adapted for the genus *Branchiostoma*. This implies that, although most of the terms can also be applied to the two other genera, it will be necessary to update AMPHX in the future to include terms specific for the *Epigonichthys* and *Asymmetron* genera.

The final objective of AMPHX is to allow the research community to use this partonomic definition of anatomical and developmental entities to assign gene expression patterns and phenotypic descriptions to each structure and developmental stage. A future objective of the AMPHX ontology should be its use as an interoperable ontology allowing comparisons between different amphioxus species and between amphioxus and other metazoan groups, particularly vertebrates. In order to perform such interspecies comparisons, homologous anatomical structures, and developmental stages have to be defined between the species being compared, and synonyms between the AMPHX ontology and other ontologies thus have to be established. However, these comparisons and the definition of homologous structures are not straightforward. While comparisons among different amphioxus species and genera are certainly feasible due to the high degree of anatomical and developmental conservation within the cephalochordate subphylum, such comparisons are significantly more complicated between amphioxus and members of other metazoan clades. The concept of biological homology, defined as sharing a common ancestor, is a binary relationship (a structure is or is not homologous to another structure). However, in an ontology it is necessary to consider relationships between cell types, tissues, and organs as a continuous rather than an all-or-nothing relationship (Wagner, 1989). As such, biological homology is unfortunately an impractical concept for cross-ontology comparisons. Ontology comparisons thus usually identify synonyms, rather than homologs. In the AMPHX ontology, we have been conservative with the definition of synonyms. We only used existing terms from other ontologies as a synonym of a term in AMPHX when the homology of the compared terms is generally accepted by the scientific community. For example, the amphioxus gastrula period (AMPHX:0000011) is homologous to the gastrula stage in UBERON (UBERON:0000109), which is defined as: “*A stage defined by complex and coordinated series of cellular movements that occurs at the end of cleavage during embryonic development of most animals. The details of gastrulation vary from species to species, but usually result in the formation of the three primary germ layers, ectoderm, mesoderm, and endoderm*”. Given that this UBERON definition allows the inclusion of slight differences in the gastrulation process between different animals, the use of this term as a synonym to the AMPHX gastrulation period was validated. Another example for a synonymous term is the amphioxus neural plate (AMPHX:1000048), which corresponds to the identical term in UBERON (UBERON:0003075), which is defined as: “*A region of embryonic ectodermal cells that lies directly above the notochord. During neurulation, they change shape and produce an infolding of the neural plate (the neural fold) that then seals to form the neural tube. The earliest recognizable dorsal ectodermal primordium of the central nervous system present near the end of gastrulation before infolding to form the neural keel; consists of a thickened pseudostratified epithelium.*”

Having been conservative with the definition of synonyms, some structures that share a common ancestor and are thus homologous have not been assigned synonyms in the AMPHX ontology. This is the case, for example, for amphioxus and vertebrate somites. Given that the derivatives of amphioxus and vertebrate somites are not identical (Gilbert, 2003; Mansfield et al., 2015; Aldea et al., 2019), the term somite is only partially synonymous in the context of a cross-ontology comparison between amphioxus and vertebrates. We thus preferred not to define somites as synonyms in AMPHX. This highlights an important aspect of the AMPHX ontology, its potential for being amended and improved by the community. In particular, the definitions of cell types, tissues, and organs in juveniles and adults are lagging behind those of embryos and larvae. The number of structures in juveniles and adults is currently considerably lower than that for the embryonic and larval stages. This seems counterintuitive, as an adult should possess a significantly higher number of anatomical structures than an embryo. This bias is due to the fact that the list of terms in the AMPHX ontology is based mostly on the existing literature, which is heavily focused toward studies of embryo and larval stages. To expand further the AMPHX ontology, it would thus be interesting to focus more attention on the characterization of the ultrastructure of amphioxus juveniles and adults.

In conclusion, we encourage the amphioxus community and other scientific communities interested in animal evolution to use the AMPHX ontology. Input and support from users will be crucial to update and improve AMPHX by adding and redefining terms as new data become available. Of particular immediate importance will be the addition of information for the underrepresented juvenile and adult stages. Data from single-cell studies could be very useful for the refinement of AMPHX through the inclusion of new cell types, and a more detailed definition of the cell types and tissues that constitute amphioxus at any given stage.

## Data Availability Statement

The datasets presented in this study can be found in online repositories. The names of the repository/repositories and accession number(s) can be found in the article/ Supplementary Material.

## Author Contributions

HE and SB created the initial version of AMPHX. All the authors corrected and updated the initial version of AMPHX. DD, VD, and NM transformed the ontology into OWL and released workflows through GitHub. HE wrote the manuscript. All authors read and approved the manuscript.

## Conflict of Interest

The authors declare that the research was conducted in the absence of any commercial or financial relationships that could be construed as a potential conflict of interest. The reviewer IK declared a past collaboration with one of the authors J-KY to the handling editor.

## Footnotes

1 https://github.com/EBISPOT/webulous

2 https://github.com/EBISPOT/amphx_ontology

3 http://obofoundry.org/ontology/amphx.html

4 http://www.ontobee.org/ontology/AMPHX

5 https://www.ebi.ac.uk/ols/ontologies/amphx

6 http://bioportal.bioontology.org/ontologies/AMPHX?p=summary

7 https://en.wikipedia.org/wiki/FAIR_data

8 https://fairsharing.org/bsg-s001582/

9 http://purl.obolibrary.org/obo/cl.owl

10 http://purl.obolibrary.org/obo/ro.owl

11 https://www.youtube.com/watch?v=p8XF7yJvW4E

## References

[B1] Acemel R. D. Tena J. J. Irastorza-Azcarate I. Marlétaz F. Gómez-Marín C. de la Calle-Mustienes E. (2016). A single three-dimensional chromatin compartment in amphioxus indicates a stepwise evolution of vertebrate Hox bimodal regulation. *Nat. Genet.* 48 336–341. 10.1038/ng.3497 26829752

[B2] Aldea D. Subirana L. Keime C. Meister L. Maeso I. Marcellini S. (2019). Genetic regulation of amphioxus somitogenesis informs the evolution of the vertebrate head mesoderm. *Nat. Ecol. Evol.* 3 1233–1240. 10.1038/s41559-019-0933-z 31263232

[B3] Bertrand S. Escriva H. (2011). Evolutionary crossroads in developmental biology: amphioxus. *Development* 138 4819–4830. 10.1242/dev.066720 22028023

[B4] Carvalho J. E. Lahaye F. Yong L. W. Croce J. C. Escrivá H. Yu J.-K. (2021). An updated staging system for cephalochordate development: one table suits them all. *Front. Cell Dev. Biol.* 9:668006. 10.3389/fcell.2021.668006 PMC8174843 34095136

[B5] Côté R. G. Jones P. Martens L. Apweiler R. Hermjakob H. (2008). The ontology lookup service: more data and better tools for controlled vocabulary queries. *Nucleic Acids Res.* 36 W372–W376. 18467421 10.1093/nar/gkn252 PMC2447739

[B6] Dehal P. Boore J. L. (2005). Two rounds of whole genome duplication in the ancestral vertebrate. *PLoS Biol.* 3:e314. 10.1371/journal.pbio.0030314 16128622 PMC1197285

[B7] Escriva H. (2018). My favorite animal, amphioxus: unparalleled for studying early vertebrate evolution. *Bioessays* 40:e1800130. 10.1002/bies.201800130 30328120

[B8] Gilbert S. F. (2003). *Developmental Biology*, 7th Edn. Sunderland, MA: Sinauer Associates, Inc.

[B9] Hirakow R. Kajita N. (1990). An electron microscopic study of the development of amphioxus, *Branchiostoma belcheri tsingtauense*: cleavage. *J. Morphol.* 203 331–344. 10.1002/jmor.1052030308 29865721

[B10] Hirakow R. Kajita N. (1991). Electron microscopic study of the development of amphioxus, *Branchiostoma belcheri tsingtauense*: the gastrula. *J. Morphol.* 207 37–52. 10.1002/jmor.1052070106 29865496

[B11] Hirakow R. Kajita N. (1994). Electron microscopic study of the development of Amphioxus, *Branchiostoma belcheri tsingtauense*: the neurula and larva. *Kaibogaku Zasshi* 69 1–13. 8178614

[B12] Holland N. D. Holland L. Z. (2010). Laboratory spawning and development of the Bahama lancelet, *Asymmetron lucayanum* (*Cephalochordata*): fertilization through feeding larvae. *Biol. Bull.* 219 132–141. 10.1086/bblv219n2p132 20972258

[B13] Holland N. D. Somorjai I. M. L. (2020). Tail regeneration in a cephalochordate, the Bahamas lancelet, *Asymmetron lucayanum*. *J. Morphol*. 282 217–229. 10.1002/jmor.21297 33179804

[B14] Hotta K. Dauga D. Manni L. (2020). The ontology of the anatomy and development of the solitary ascidian *Ciona*: the swimming larva and its metamorphosis. *Sci. Rep.* 10:17916. 10.1038/s41598-020-73544-9 PMC7578030 33087765

[B15] Jackson R. C. Balhoff J. P. Douglass E. Harris N. L. Mungall C. J. Overton J. A. (2019). ROBOT: a tool for automating ontology workflows. *BMC Bioinformatics* 20:407. 10.1186/s12859-019-3002-3 PMC6664714 31357927

[B16] Jupp S. Burdett T. Leroy C. Parkinson H. (2015). “A new ontology lookup service at EMBL-EBI,” in *Proceedings of the SWAT4LS International Confernce* (Cambridge: SWAT4LS), 118–119.

[B17] Lee R. Y. Sternberg P. W. (2003). Building a cell and anatomy ontology of *Caenorhabditis elegans*. *Comp. Funct. Genomics* 4 121–126. 10.1002/cfg.248 18629098 PMC2447384

[B18] Manni L. Gasparini F. Hotta K. Ishizuka K. J. Ricci L. Tiozzo S. (2014). Ontology for the asexual development and anatomy of the colonial chordate *Botryllus schlosseri*. *PLoS One* 9:e96434. 10.1371/journal.pone.0096434 24789338 PMC4006837

[B19] Mansfield J. H. Haller E. Holland N. D. Brent A. E. (2015). Development of somites and their derivatives in amphioxus, and implications for the evolution of vertebrate somites. *Evodevo* 6:21. 10.1186/s13227-015-0007-5 PMC4458041 26052418

[B20] Marletaz F. Firbas P. N. Maeso I. Tena J. J. Bogdanovic O. Perry M. (2018). Amphioxus functional genomics and the origins of vertebrate gene regulation. *Nature* 564 64–70. 30464347 10.1038/s41586-018-0734-6 PMC6292497

[B21] Mungall C. J. Torniai C. Gkoutos G. V. Lewis S. E. Haendel M. A. (2012). Uberon, an integrative multi-species anatomy ontology. *Genome Biol.* 13:R5. 10.1515/jib-2007-65 PMC3334586 22293552

[B22] Noy N. F. Shah N. H. Whetzel P. L. Dai B. Dorf M. Griffith N. (2009). BioPortal: ontologies and integrated data resources at the click of a mouse. *Nucleic Acids Res.* 37 W170–W173. 19483092 10.1093/nar/gkp440 PMC2703982

[B23] Ong E. Xiang Z. Zhao B. Liu Y. Lin Y. Zheng J. (2017). Ontobee: A linked ontology data server to support ontology term dereferencing, linkage, query and integration. *Nucleic Acids Res.* 45 D347–D352. 27733503 10.1093/nar/gkw918 PMC5210626

[B24] Paris M. Escriva H. Schubert M. Brunet F. Brtko J. Ciesielski F. (2008). Amphioxus postembryonic development reveals the homology of chordate metamorphosis. *Curr. Biol.* 18 825–830. 10.1016/j.cub.2008.04.078 18514519

[B25] Putnam N. H. Butts T. Ferrier D. E. Furlong R. F. Hellsten U. Kawashima T. (2008). The amphioxus genome and the evolution of the chordate karyotype. *Nature* 453 1064–1071. 18563158 10.1038/nature06967

[B26] Sansone S. A. McQuilton P. Rocca-Serra P. Gonzalez-Beltran A. Izzo M. Lister A. L. (2019). FAIRsharing as a community approach to standards, repositories and policies. *Nat. Biotechnol.* 37 358–367. 10.1038/s41587-019-0080-8 30940948 PMC6785156

[B27] Segerdell E. Bowes J. B. Pollet N. Vize P. D. (2008). An ontology for *Xenopus* anatomy and development. *BMC Devel. Biol.* 8:92. 10.1186/1471-213X-8-92 18817563 PMC2561031

[B28] Smith B. Ashburner M. Rosse C. Bard J. Bug W. Ceusters W. (2007). The OBO foundry: coordinated evolution of ontologies to support biomedical data integration. *Nat. Biotechnol.* 25 1251–1255. 10.1038/nbt1346 17989687 PMC2814061

[B29] Somorjai I. M. Somorjai R. L. Garcia-Fernandez J. Escriva H. (2012). Vertebrate-like regeneration in the invertebrate chordate amphioxus. *Proc. Natl. Acad. Sci. U.S.A.* 109 517–522. 10.1073/pnas.1100045109 22203957 PMC3258630

[B30] Van Slyke C. E. Bradford Y. M. Westerfield M. Haendel M. A. (2014). The zebrafish anatomy and stage ontologies: representing the anatomy and development of *Danio rerio*. *J. Biomed. Semantics* 5:12. 10.1186/2041-1480-5-12 24568621 PMC3944782

[B31] Wagner G. P. (1989). The biological homology concept. *Annu. Rev. Ecol. Syst.* 20 51–69. 10.1146/annurev.es.20.110189.000411

[B32] Yuan S. Ruan J. Huang S. Chen S. Xu A. (2015). Amphioxus as a model for investigating evolution of the vertebrate immune system. *Dev. Comp. Immunol.* 48 297–305. 10.1016/j.dci.2014.05.004 24877655

